# Increased levels of the synaptic proteins PSD-95, SNAP-25, and neurogranin in the cerebrospinal fluid of patients with Alzheimer’s disease

**DOI:** 10.1186/s13195-022-01002-x

**Published:** 2022-04-23

**Authors:** Pia Kivisäkk, Becky C. Carlyle, Thadryan Sweeney, James P. Quinn, Christopher E. Ramirez, Bianca A. Trombetta, Muriel Mendes, Mary Brock, Carrie Rubel, Julie Czerkowicz, Danielle Graham, Steven E. Arnold

**Affiliations:** 1 Department of Neurology, Alzheimer’s Clinical and Translational Research Unit, Massachusetts General Hospital, 114 16th Street, Room 2300, Charlestown, MA 02129 USA; 2 Quanterix Corporation, Billerica, MA USA; 3 Biogen, Cambridge, MA USA

**Keywords:** Alzheimer’s disease, Biomarkers, Cerebrospinal fluid, Synaptic markers, PSD-95, SNAP-25, Neurogranin, Neurodegenerative diseases

## Abstract

**Background:**

There is currently a lack of reliable and easily accessible biomarkers predicting cognitive decline in Alzheimer’s disease (AD). Synaptic dysfunction and loss occur early in AD and synaptic loss measured in the brain tissue and by PET are closely linked to cognitive decline, rendering synaptic proteins a promising target for biomarker development.

**Methods:**

We used novel Simoa assays to measure cerebrospinal fluid (CSF) levels of two synaptic biomarker candidates, postsynaptic density protein 95 (PSD-95/DLG4), and the presynaptically localized synaptosomal-associated protein 25 (SNAP-25), as well as neurogranin (Ng), an established postsynaptic biomarker. CSF samples from two well-characterized cohorts (*n*=178 and *n*=156) were selected from banked samples obtained from diagnostic lumbar punctures containing subjects with amyloid-ß (Aß) positive AD, subjects with non-AD neurodegenerative diseases, subjects with other neurological conditions, and healthy controls (HC).

**Results:**

All subjects had detectable CSF levels of PSD-95, SNAP-25, and Ng. CSF levels of PSD-95, SNAP-25, and Ng were all correlated, with the strongest correlation between the presynaptic SNAP-25 and the postsynaptic neurogranin. AD subjects had on average higher concentrations of all three synaptic markers compared to those with non-AD neurodegenerative diseases, other neurological disorders, and HCs. Increased CSF levels of PSD-95, SNAP-25, and Ng were, however, not specific for AD and were present in sporadic cases with inflammatory or vascular disorders as well. High CSF levels of PSD-95 were also observed in a few subjects with other neurodegenerative disorders.

**Conclusion:**

The data establishes PSD-95 as a promising CSF marker for neurodegenerative disease synaptic pathology, while SNAP-25 and Ng appear to be somewhat more specific for AD. Together, these synaptic markers hold promise to identify early AD pathology, to correlate with cognitive decline, and to monitor responses to disease-modifying drugs reducing synaptic degeneration.

**Supplementary Information:**

The online version contains supplementary material available at 10.1186/s13195-022-01002-x.

## Background

Synaptic dysfunction and loss occur early in Alzheimer’s disease (AD) and may be present before a reduction in neuronal density is apparent [1–3]. While established cerebrospinal fluid (CSF) AD biomarkers such as amyloid-ß42 (Aß42) and phospho-tau (pTau181) have proven to be of diagnostic value in AD, they are less predictive of cognitive decline, and it is unclear how well their levels reflect clinically relevant changes during disease progression or treatment trials. Synaptic loss measured by various methods such as electron microscopy, neurochemical measurements of synaptic protein concentrations, and PET, has been reported to better correlate with cognitive decline in AD in multiple studies [4, 5], rendering synaptic proteins a promising target for biomarker development. Increased levels of both pre- and post-synaptic proteins including synaptosomal-associated protein 25 (SNAP-25), growth-associated protein 43 (GAP-43), synaptotagmin-1, and neurogranin (Ng) have been detected in the CSF in the early stages of AD presumably due to release from degenerating synapses [6]. Here, we used two novel pre-commercial Simoa assays developed by Quanterix (Billerica, MA) to measure CSF levels of postsynaptic density protein 95 (PSD-95), a synaptic protein not previously described in the CSF, and SNAP-25, an emerging synaptic biomarker of interest, in subjects with AD, non-AD neurodegenerative diseases, other neurological conditions, and healthy controls. The CSF Aß42/40 ratio was used to establish AD as the underlying pathophysiology for mild cognitive impairment (MCI) and dementia. In addition, levels of Ng, an established synaptic marker, were quantified using a commercially available ELISA assay (Euroimmun, Lübeck, Germany) for comparison purposes.

PSD-95 (also known as SAP-90 or DLG4) is a scaffolding protein located at the postsynaptic density of excitatory synapses which binds directly or indirectly to NMDA and AMPA receptors, potassium channels, and associated signaling proteins resulting in their clustering during the formation and maintenance of dendritic spines [7]. Altered PSD-95 expression modulates receptor retention at the synapse and PSD-95 is an important factor in synaptic plasticity and the stabilization of synaptic changes during long-term potentiation [8]. Animal studies suggest that PSD-95 disruption is associated with cognitive and learning deficits [9]. Reduced expression of PSD-95 has been observed in brain tissue from AD subjects [10, 11] and in mouse models of AD [11].

SNAP-25 is a component of the presynaptic SNARE complex, which mediates the fusion and exocytosis of synaptic vesicles in neurons and the subsequent neurotransmitter release [12]. CSF levels of SNAP-25 have previously been shown to be elevated in AD subjects using mass spectrometry [13] or a microparticle-based immunoassay on the Singulex Erenna system [14–16]. In subjects carrying autosomal-dominant AD mutations, SNAP-25 CSF levels were increased 15–19 years prior to symptom onset [14], correlated with cognitive measures [14, 15], and predicted clinical decline in subjects with MCI [15].

Our results showed that subjects with AD on average had markedly increased PSD-95 and SNAP-25 CSF levels compared to both healthy controls and subjects with other neurodegenerative diseases, further promoting these two proteins as biomarkers for synaptic dysfunction in AD.

## Methods

### Study subjects

Two independent cohorts of CSF samples were selected from the Mass General Institute for Neurodegenerative Disease (MIND) biorepository, which consists of CSF samples collected from diagnostic lumbar punctures at the Department of Neurology at Massachusetts General Hospital. CSF samples from healthy research participants were obtained from the LifeSPAN biorepository. Clinical diagnoses were verified in chart review by an experienced neurologist (SEA), and AD status was verified by the CSF Aß42/40 ratio measured by Quanterix (Cohort I) or Euroimmun (Cohort II) immunoassays as described below.

The initial cohort (*n*=178) consisted of subjects with AD (*n*=37), subjects with a range of other neurodegenerative (NeuroDegen; *n*=62) or non-neurodegenerative (NeuroCtrl; *n*=59) neurological diseases, and healthy controls (HC; *n*=20). The validation cohort (*n*=165) consisted of a larger number of AD subjects (*n*=99) with varying levels of cognitive impairment, subjects with NeuroDegen diseases (*n*=6), NeuroCtrls (*n*=33), and HC (*n*=21). Further demographic and diagnostic details can be found in Table 1.

**Table 1:** Clinical and demographic information

		Diagnostic Groups	*N*	Age		Gender	Aß42/40 ratio^d^	pTau181	tTau	NfL	GFAP
				Mean (SD)	Range	Females *N* (%)	Mean (SD)	Mean (SD)	Mean (SD)	Mean (SD)	Mean (SD)
Cohort I	AD	AD MCI	18	71.4 (6.5)	59–84	9 (50%)	0.044 (0.010)	95.4 (38.5)	460 (120)	1224 (697)	19,811 (7348)
		AD dementia	19	70.3 (9.5)	56–84	7 (37%)	0.048 (0.010)	93.3 (40.7)	428 (153)	1422 (483)	19,489 (6319)
	NeuroDegen	Frontotemporal dementia (FTD)	18	65.7 (8.2)	51–79	7 (39%)	0.080 (0.015)	32.4 (17.0)	236 (95)	2408 (2648)	17,207 (6923)
		Lewy body dementia (LBD/Park)	14	71.6 (11.2)	48–88	4 (29%)	0.081 (0.019)	31.1 (16.6)	227 (105)	2292 (3449)	18,005 (6510)
		Motor neuron disease (MND)	6	55.7 (11.1)	40–65	3 (50%)	0.083 (0.011)	26.4 (9.4)	179 (76)	6046 (8140)	9864 (6463)
		Dementia NOS	12	75.5 (8.3)	58–89	8 (67%)	0.097 (0.017)	21.7 (8.8)	187 (114)	1733 (1356)	20,663 (14,610)
		MCI NOS	12	73.3 (8.6)	60–86	3 (75%)	0.080 (0.012)	27.5 (9.2)	232 (82)	1466 (1444)	19,176 (8624)
	NeuroCtrl	Immune/demyelinating diseases (INFL)^a^	20	47.5 (12.8)	24–69	9 (45%)	0.082 (0.011)	30.7 (33.1)	283 (224)	3829 (7812)	11,337 (6400)
		Idiopathic intracranial hypertension (IIH)	20	43.3 (12.5)	20–71	14 (70%)	0.085 (0.009)	29.2 (16.8)	197 (114)	362 (155)	7602 (3001)
		Controls with other neurological diseases^b^	19	66.5 (8.5)	55–85	10 (53%)	0.093 (0.020)	30.0 (11.6)	234 (87)	4373 (9889)	14,691 (7043)
	HC	Healthy controls	20	54.4 (14.5)	23–77	9 (45%)	0.087 (0.010)	30.0 (9.5)	204 (71)	654 (299)	11,751 (6691)
Cohort II	AD	AD asymptomatic^c^	6	69.0 (13.3)	56–87	2 (33%)	0.054 (0.015)	73.4 (27.3)	468 (115)	n.d.	n.d.
		AD MCI	57	67.1 (8.6)	51–82	25 (44%)	0.047 (0.012)	107.7 (36.2)	510 (192)	n.d.	n.d.
		AD dementia	42	69.3 (9.4)	51–89	18 (43%)	0.047 (0.013)	130.8 (47.7)	615 (259)	n.d.	n.d.
	NeuroDegen	Other neurodegenerative diseases	6	66.5 (11.4)	46–79	2 (33%)	0.118 (0.022)	24.8 (6.4)	211 (31)	n.d.	n.d.
	NeuroCtrl	Controls with other neurological diseases	33	58.3 (14.8)	25–84	18 (55%)	0.116 (0.018)	22.7 (8.0)	172 (49)	n.d.	n.d.
	HC	Healthy controls	21	58.5 (15.5)	21–85	10 (48%)	0.124 (0.026)	28.7 (12.8)	212 (85)	n.d.	n.d.

^a^Immune/demyelinating diseases: multiple sclerosis (8), meningitis (3), CNS vasculitis (2), neurosarcoidosis (2), demyelinating polyneuropathy (2), pleocytosis NOS (2), autoimmune encephalopathy (1)

^b^Other neurological controls: cranial neuropathies (4), vascular (3), headaches (3), normal pressure hydrocephalus (3), polyneuropathy (3), nystagmus (2), essential tremor (1)

^c^AD asymptomatic: cognitively unimpaired subjects with positive CSF AD biomarkers

^d^Aß42/40 ratio was measured by Quanterix Neuro 4-plex E (cohort I) or Euroimmun beta-amyloid (1-40) and (1-42) ELISA assays (cohort II)

*AD* Alzheimer’s disease, *MCI* mild cognitive impairment, *NeuroDegen* non-AD neurodegenerative diseases, *NeuroCtrl* other neurological conditions, *HC* healthy controls, *NOS* not otherwise specified

### CSF sampling and analysis

Selected samples were collected between 2014 and 2020. Samples were collected and aliquoted in polypropylene plasticware, frozen on dry ice within 30 min of collection, and stored at −80 °C until use. PSD-95 and SNAP-25 concentrations were measured by the Quanterix Accelerator lab (Billerica, MA) using pre-commercial research assays. The PSD-95 assay used a monoclonal mouse IgG anti-PSD-95 antibody as the capture antibody and a monoclonal rabbit IgG anti-PSD-95 antibody as the detection antibody. The specificity of the antibodies was verified using Western blot of human brain homogenates which showed bands at the expected molecular weights of 80 and 95 kDa (Supplementary Figure 1). The SNAP-25 assay, which targets the soluble N-terminal fragment of SNAP-25 (aa 2-47), has since become commercially available (Quanterix Simoa SNAP-25 Advantage Kit). It uses a monoclonal mouse antibody against the full-length unaltered SNAP-25 protein as the capture antibody and a mouse monoclonal antibody against a recombinant form of SNAP-25 protein with the epitope mapped to the N-terminal region as the detection antibody. The sensitivity of the PSD-95 and SNAP-25 assays was estimated by their LLOD (defined as mean+2SD of the blank), and LLOQ. LLOQ was defined for the SNAP-25 assay as the lowest dilution with a CV of 20% and recovery between 80 and 120% of the expected concentration and for the PSD-95 assay based on CV profiling, which employs a fixed signal CV to estimate the associated concentration CV where LLOQ is the concentration at which the calibration curve intersects the CV curve at 20% CV. Dilution linearity was evaluated by spiking recombinant protein into the target matrix, then diluting in sample buffer and calculating the percent recovery at each level against the baseline dilution. The spike recovery was defined as percent recovery of spiked protein at final dilution. Aß40, Aß42, NfL, and GFAP were measured in Cohort I by the Quanterix Accelerator lab using the Quanterix Simoa Neurology 4-plex E (N4PE) assay. Aß40 and Aß42 were measured in Cohort II using Euroimmun Beta-Amyloid (1-40) and (1-42) ELISA assays (Lübeck, Germany) on a semiautomated Tecan Freedom Evo liquid handler (Männedorf, Switzerland) in the Arnold lab following manufacturer’s instructions. Euroimmun Total-Tau, phospho-Tau/pTau(181), and Neurogranin (Trunc P75) ELISA assays were also performed in the Arnold lab on the Tecan platform. 

### Statistical analysis

Biomarker concentrations were log-transformed to satisfy assumptions of normality of their distribution. Correlation analyses were performed using Pearson correlation and linear regression in STATA/SE version 16.1. Differences between diagnostic groups were evaluated using ANOVA and Tukey’s Honest Significant Difference test adjusting for age, sex, and the biomarker in question. The subgroup analysis between different AD subsets was performed using weighted logistic regression, and multiple testing correction was performed using the Benjamini-Hochberg method. To assess the classification utility of the biomarkers, area under the curve (AUC) values were computed using a logistic regression model fit to subsets of the dataset proved significant by the previous procedures [17]. To control for the effect of age, biomarker levels were residualized on age for the logistic regression models. The above procedures were carried out using the R statistical software version 4.0.4. Statistical analysis to determine differences in synaptic markers between the different neurodegenerative and non-neurodegenerative neurological diseases was not performed due to the large number of comparisons and small sample sizes. This data is only presented to provide information on outlier status. Values in graphs are presented without log transformation for visualization purposes.

## Results

### Patient characteristics

Two independent cohorts were used to identify and validate between-group differences in CSF levels of PSD-95 and SNAP-25. The initial cohort (*n*=178) was selected to contain subjects with a range of neurological diseases (including AD and other neurodegenerative and non-neurodegenerative neurological diseases) and HCs and to span a very broad spectrum of ages from young to old (20–89 years). The validation cohort (*n*=165) was selected to contain a larger number of AD subjects with varying levels of cognitive impairment. The preliminary analysis showed that there were no effects of age (PSD-95 - cohort 1: *r*=0.13; cohort 2: *r*=0.24; SNAP-25 - cohort 1: *r*=−0.027; Cohort 2: *r*=−0.01; p=n.s. for all comparisons) or gender (data not shown) on levels of PSD-95 nor SNAP-25. All AD subjects had a low CSF Aß42/40 ratio to establish AD as the underlying pathophysiology for MCI and dementia (Supplementary Figure 2).

### PSD-95 and SNAP-25 assay performance

Bead-based digital Simoa immunoassays were developed to detect SNAP-25 and PSD-95 in human CSF. Conditions were optimized for maximal sensitivity and analytical performance. The assays were evaluated for sensitivity, dilution linearity, spike & recovery, and analyte detectability in normal human CSF. The SNAP-25 assay had an LLOD of 0.88 pg/ml and an LLOQ of 2.56 pg/ml, which resulted in a functional LLOQ of 10.24 pg/ml after multiplying by the dilution factor (4x). The quantifiability was >80% of normal samples above LLOQ, the linearity of sample dilution was 80–124% in CSF, and the recovery of sample spike was 95–110%. The PSD-95 assay had an LLOD of 0.5 pg/ml and an LLOQ of 1.0 pg/ml, resulting in a functional LLOQ of 8.0 pg/ml at 8x dilution. The quantifiability was 100% of normal samples above LLOQ, the linearity of sample dilution was 90–113% in CSF, and the recovery of sample spike was 84–100%.

Concentrations of PSD-95 and SNAP-25 were above the LLOQ in all subjects of the two cohorts included in the current study. The samples were run in duplicate with intraplate coefficients of variation (CVs) of 3.2±2.5% (mean±SD) for the PSD-95 assay and 1.9±1.8% for the SNAP-25 assay. The initial and validation cohorts were analyzed 6 months apart using different reagent lots for the PSD-95 and SNAP-25 assays. The two reagent lots were manually prepared and did not go through commercial lot-to-lot testing, which resulted in a large lot-to-lot variability for PSD-95. Identical bridge samples repeated in both lots showed a mean concentration of 283±111 pg/ml (mean±SD) in Lot 1 and 138±66 pg/ml in Lot 2 (CV: 50.2±9.7%). The results between the two batches were, however, tightly correlated (*r*=0.96; *p*<0.05) demonstrating that the differences were due to a constant bias. The difference between the two batches for SNAP-25 were smaller (Lot 1: 94±24 pg/ml; Lot 2: 81±18 pg/ml; CV: 10.5±2.7%) and also tightly correlated (*r*=0.99; *p*<0.005).

### Correlations among CSF PSD-95, SNAP-25, and neurogranin (Ng) levels

**Fig. 1:**
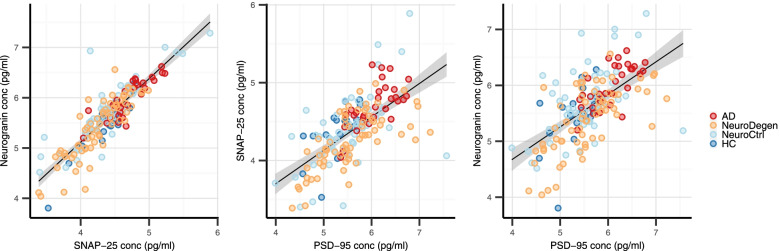
Correlations between CSF levels of PSD-95, SNAP-25, and neurogranin stratified by diagnostic group

CSF levels of all three synaptic markers were highly correlated with each other with the strongest correlation between SNAP-25 and Ng (all subjects: *r*=0.87; *p*<0.0001; Fig 1A). The subgroup analysis showed that this correlation was present in all diagnostic groups at similar strength (*r*=0.82–0.88; *p*<0.0001 for all comparisons; Supplementary Table 1). SNAP-25 and Ng were also significantly correlated with PSD-95, although these correlations were less strong (all subjects: *r*=0.44 and *r*=0.41, respectively; *p*<0.0001 for both comparisons; Fig 1B, C). The subgroup analysis showed a few NeuroCtrl and NeuroDegen subjects who had high PSD-95 levels without correspondingly high levels of SNAP-25 or Ng and vice versa resulting in weaker correlations in these groups compared to the other diagnostic groups resulting in the overall less strong correlations (Supplementary Table 1).

### Subjects with AD had higher CSF levels of PSD-95, SNAP-25, and Ng

**Fig. 2:**
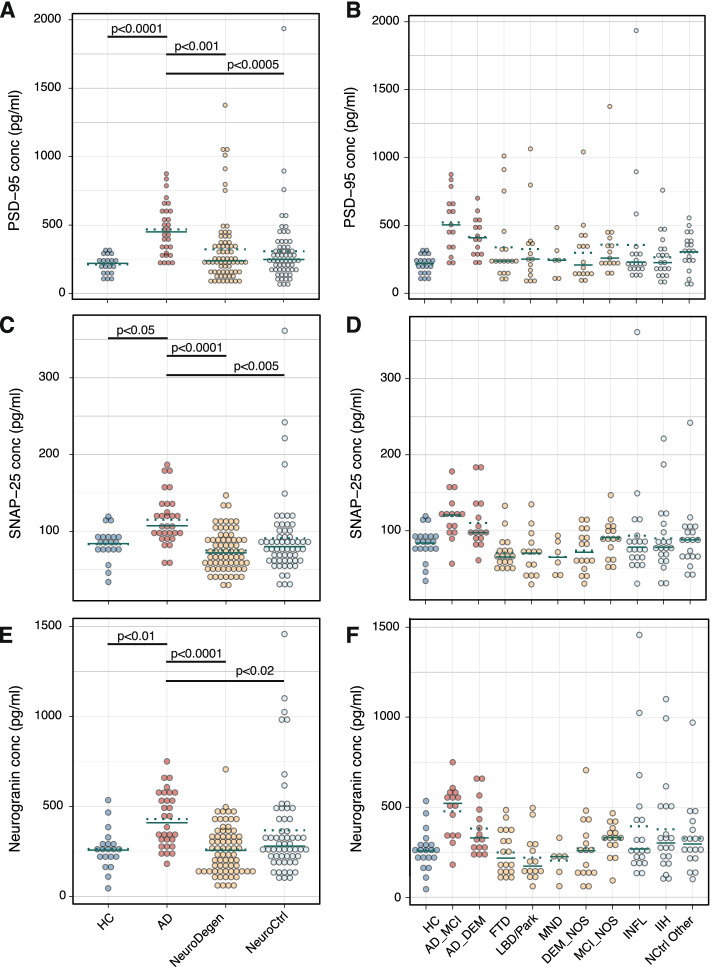
CSF levels of PSD-95 (**A**), SNAP-25 (**C**), and Neurogranin (**E**) in healthy controls (HC) and subjects with Alzheimer’s disease (AD), non-AD neurodegenerative diseases (NeuroDegen), and neurological controls (NeuroCtrl) from cohort I. CSF levels of PSD-95 (**B**), SNAP-25 (**D**), and neurogranin (**F**) by diagnosis. Solid green lines represent the median of the groups and dotted green lines represent the mean. Statistical analysis to determine differences between the individual NeuroDegen and NeuroCtrl diseases (**B**, **D**, and **F**) was not performed due to the large number of comparisons and small sample sizes, and graphs are included to provide information on outlier status

Between-group differences in synaptic markers in the initial cohort were determined using ANOVA followed by Tukey’s post hoc test (Supplementary Table 2A). This demonstrated that CSF levels of PSD-95 on average were significantly higher in subjects with AD (452±176 pg/ml; Fig. 2A) compared to subjects with NeuroDegen diseases (316±271 pg/ml; *p*<0.001), NeuroCtrls (308±268 pg/ml; *p*<0.0005), and HC (213±70 pg/ml; *p*<0.0001). However, high PSD-95 levels were not specific for AD and subjects with very high PSD-95 levels (defined manually as >650 pg/ml based on levels exceeding the main PSD-95 distribution) were observed in 7 of 62 subjects (11%) with NeuroDegen diseases [frontotemporal dementia (*n*=3), Lewy body dementia (*n*=2), and dementia/MCI NOS (*n*=2)] and in 3 of 59 subjects (5%) with other neurological diseases [CNS vasculitis and multiple strokes (*n*=1), neurosarcoidosis (*n*=1), and idiopathic intracranial hypertension (IIH; *n*=1); Fig. 2B]. The highest PSD-95 level was observed in the subject with CNS vasculitis and multiple strokes (1935 pg/ml).

SNAP-25 CSF levels were similarly higher in subjects with AD (114±30 pg/ml; Fig. 2C) compared to subjects with NeuroDegen diseases (71±24 pg/ml; *p*<0.0001), NeuroCtrls (91±53 pg/ml; *p*<0.005), and HC (83±21 pg/ml; *p*<0.05). High SNAP-25 levels (defined manually as >150 pg/ml) were not observed in any NeuroDegen subject but were found in 5 of the 59 NeuroCtrls [8%; IIH (*n*=2), Mollaret’s meningitis (*n*=1), neurosarcoidosis (*n*=1), and sinus thrombosis (*n*=1); Fig. 2D]. The highest SNAP-25 level was observed in the subject with neurosarcoidosis (361 pg/ml).

The overall profile of Ng expression was very similar to that of SNAP-25 consistent with the high correlation between the two markers. Ng CSF levels were higher in subjects with AD (418±142 pg/ml; Fig. 2E) compared to subjects with NeuroDegen diseases (248±133 pg/ml; *p*<0.0001), NeuroCtrls (367±270 pg/ml; *p*<0.02), and HC (264±111 pg/ml; *p*<0.01). The same 5 controls with high SNAP-25 levels were also exceeding the cutoff for high Ng levels (manually defined as >600 pg/ml), again with the highest levels observed in the subject with neurosarcoidosis (1457 pg/ml; Fig. 2F). Two additional NeuroCtrls [meningitis (*n*=1) and IIH (*n*=1)] and one subject with NeuroDegen diseases (dementia/MCI NOS) exceeded the Ng cutoff.

### Diagnostic classification model

**Fig. 3:**
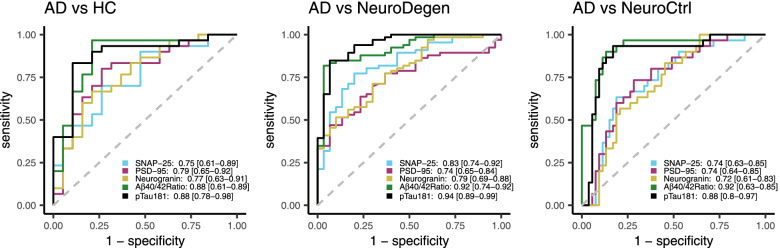
ROC-AUC curves for classification of AD vs HC (**A**), AD vs non-AD neurodegenerative diseases (**B**), and AD vs neurological controls (**C**) using PSD-95, SNAP-25, or neurogranin. The amyloid ß42/40 ratio and pTau181 are included as a reference. Graphs show AUC values with 95% confidence intervals

A logistic regression model was used to determine the ability of the synaptic markers to differentiate between AD and the control groups (Fig. 3A–C). All three synaptic markers showed a good ability to differentiate between AD and HC (Fig. 3A) with the best area under the curve (AUC) observed for PSD-95 (0.79) and marginally lower values for SNAP-25 (0.75) and Ng (0.77). The ability to discriminate between AD and NeuroDegen diseases was similar with AUC values for the three synaptic markers between 0.74 and 0.83 (Fig. 3B) and between AD and NeuroCtrls with AUC values between 0.72 and 0.74 (Fig. 3C). The performance of the synaptic markers to discriminate between AD and the control groups was, however, less than the performance of pTau181 which resulted in AUC values between 0.88-0.94 (Fig. 3A–C).

### CSF levels of PSD-95, SNAP-25, and Ng correlated with CSF levels of tTau, pTau181, and the Aß42/40 ratio, but not with NfL or GFAP

Within the AD group, CSF levels of SNAP-25 and Ng were strongly correlated with CSF levels of tTau (*r*=0.91 and 0.86, respectively; *p*<0.0001 for both comparisons) and pTau181 (*r*=0.81 and 0.69, respectively; *p*<0.0001 for both comparisons). Levels of PSD-95 also correlated with tTau (*r*=0.60; *p*<0.0001) and pTau181 (*r*=0.57; *p*<0.0002), albeit not as strongly. All three synaptic markers showed a negative correlation with the Aß42/40 ratio (*r*=−0.37 to −0.53; *p*<0.05 for all comparisons) in the subjects with AD. There were no correlations with NfL or GFAP (Table 2).

**Table 2:** Correlation between CSF levels of PSD-95, SNAP-25, or neurogranin and CSF AD biomarkers

	PSD-95			SNAP-25			Neurogranin		
	r	r2	p	r	r2	p	r	r2	p
NfL	0.08	0.01	n.s.	−0.01	0	n.s.	−0.08	0.01	n.s.
GFAP	0.06	0	n.s.	0.13	0.02	n.s.	0.08	0.01	n.s.
Aß42/40 ratio	−0.37	0.13	0.026	−0.39	0.16	0.015	−0.53	0.29	0.0007
pTau181	0.57	0.32	0.0002	0.81	0.66	<0.0001	0.69	0.47	<0.0001
tTau	0.6	0.36	0.0001	0.92	0.84	<0.0001	0.86	0.74	<0.0001

*Abbreviations*: *NfL* neurofilament light, *GFAP* glial fibrillary acidic protein, *Aß42/40 ratio* amyloid ß42/40 ratio, *pTau181* Phospho-Tau 181, *tTau* total tau

### CSF Levels of PSD-95 and SNAP-25 in the validation cohort

**Fig. 4:**
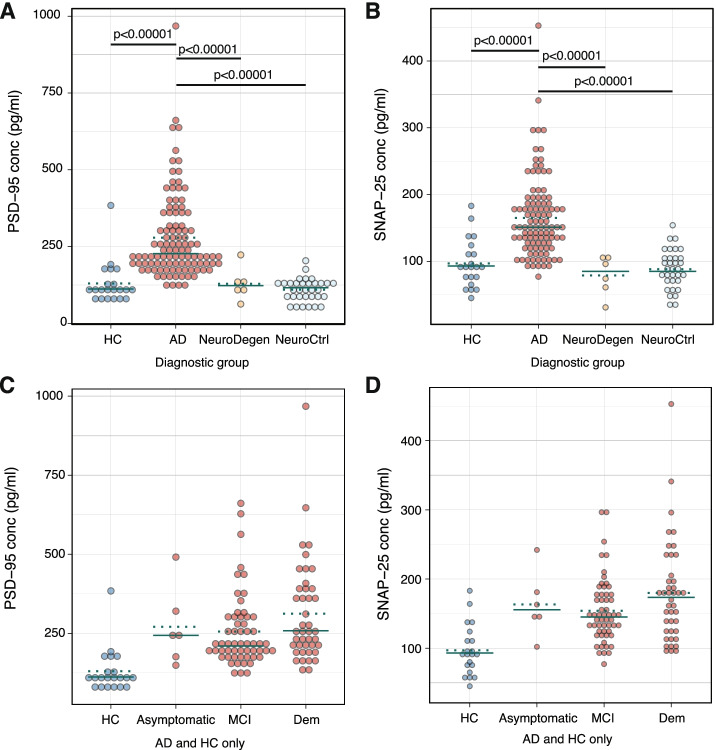
CSF levels of PSD-95 (**A**) and SNAP-25 (**B**) in healthy controls (HC) and subjects with AD, non-AD neurodegenerative diseases (NeuroDegen), and other neurological conditions (NeuroCtrl) from cohort II. PSD-95 (**C**) and SNAP-25 (**D**) levels in HC and AD subjects stratified by cognitive status. Asymptomatic refers to cognitively unimpaired subjects with positive CSF AD biomarkers. Solid green lines represent the median of the groups and dotted green lines represent mean

Next, CSF levels of PSD-95 and SNAP-25 were measured in a second, independent cohort (Fig. 4A, B; Supplementary Table 2B). Between-group analysis using ANOVA and Tukey’s post hoc test confirmed that AD subjects had higher levels of both PSD-95 (279±138 pg/ml) and SNAP-25 (163±62 pg/ml) compared to HC (PSD-95: 130±68 pg/ml; *p*<0.00001; SNAP-25: 97±36 pg/ml; *p*<0.00001), subjects with NeuroDegen diseases (PSD-95: 129±53 pg/ml; *p*<0.00001; SNAP-25: 79±30 pg/ml; *p*<0.00001), and NeuroCtrls (PSD-95: 110±38 pg/ml; *p*<0.00001; SNAP-25: 88±29 pg/ml; *p*<0.00001).

The larger number of subjects with AD in the second cohort allowed for comparisons between different AD subsets (Supplementary Table 2C). A logistic regression model with Benjamini-Hochberg correction showed that increased levels of PSD-95 and SNAP-25 were observed in early AD as both subjects with MCI and a small number of cognitively unimpaired subjects with positive CSF AD biomarkers (asymptomatic AD, *n*=6) had increased levels compared to HC (PSD-95 - HC: 130±68 pg/ml; asymptomatic AD: 270±123 pg/ml; pAdj<0.01; AD MCI: 255±116 pg/ml; pAdj<0.00001; SNAP-25 - HC: 97±36 pg/ml; asymptomatic AD: 163±47 pg/ml; pAdj<0.01; AD MCI: 151±51 pg/ml; pAdj=0.00005; Fig. 4C, D). Levels of PSD-95 and SNAP-25 were nominally higher in AD subjects with dementia (PSD-95: 312±161 pg/ml; SNAP-25: 180±73 pg/ml) compared to MCI in unadjusted analysis (*p*<0.05 for both comparisons; Fig. 4C, D), but this did not remain statistically significant after multiple testing correction.

## Discussion

We used a novel Simoa assay to quantify CSF levels of PSD-95, an important component of post-synaptic densities, in subjects with AD and related these levels to two more established synaptic markers, SNAP-25 and Ng. Our data showed increased CSF levels of all three synaptic markers in AD compared to other disease groups including other neurodegenerative diseases, inflammatory CNS conditions, CSF circulation disorders, and HC. Increased levels of the three synaptic proteins were, however, not specific to AD and sporadic subjects with high levels were also observed in the control groups. Synaptic loss is not a primary phenomenon unique to AD, but most likely a downstream phenomenon of synaptic injury or degeneration occurring in many neurological conditions. The highest levels in our study were present in subjects with inflammatory conditions such as neurosarcoidosis, vasculitis, and meningitis, where activation of microglia and astrocytes as well as infiltrating immune cells can lead to the release of factors such as glutamate and proinflammatory cytokines, triggering a neurotoxic environment [18, 19]. We did not see increased CSF levels of Ng nor SNAP-25 in subjects with other neurodegenerative diseases consistent with previous studies that have suggested that Ng is increased specifically in AD neurodegeneration and not in other neurodegenerative diseases or subjects with non-neurodegenerative cognitive impairment [20, 21]. In contrast, high CSF levels of PSD-95 were detected in 11% of subjects with non-AD neurodegenerative diseases. It is unlikely that this reflects diagnostic misclassification or a mixed AD pathology as the CSF Aß42/40 ratio was normal in these subjects. All neurodegenerative diseases are characterized by synaptic dysfunction to some extent, and it is not clear why SNAP-25 and Ng may be selectively increased in the CSF of AD subjects. Aggregation of oligomeric tau, which is present in all tauopathies including AD, can trigger synaptic damage [22]. The pattern of tau aggregates is, however, distinct in each tauopathy, and it is plausible that regional differences or a varying degree of synaptic damage can lead to different concentrations of synaptic proteins in the CSF. Aggregated α-synuclein, characteristic for the group of α-synucleinopathies, can similarly trigger synaptic damage in dopaminergic neurons [23].

While we observed a tight correlation between SNAP-25 and Ng, the level of correlation between PSD-95 and the two other synaptic markers was less pronounced. It is interesting that the two proteins with the highest degree of correlation in this study are localized at different synaptic compartments, while there was greater dissociation between the two post-synaptic proteins. It is known that there is a differential involvement of synaptic components in AD brains [24], and while CSF levels of synaptic markers often track together [25], there have also been reports of a lack of correlation [26, 27]. Further studies are needed to understand if a panel of synaptic markers reflecting different aspects of synaptic dysfunction provides additive information over an individual protein as well as if any synaptic protein is a better predictor of cognitive decline than others. It is also possible that PSD-95’s distinctive profile reflects greater sensitivity of its assay for the extent or rate of synaptic injury across diseases than SNAP-25 or Ng.

Previous studies have repeatedly demonstrated that CSF levels of Ng and SNAP-25 correlate with cognitive measures and that increased CSF levels of Ng and SNAP-25 are present already in early AD or even before symptom onset [5, 6]. We utilized a cohort of CSF samples collected from a lumbar puncture service within a general neurology clinic to include a wide range of diagnostic groups, but most cases did not have standardized cognitive testing proximate to their LPs nor consistent longitudinal follow-up as these subjects were referred from many different providers. Our cohort included a few AD cases with positive CSF AD biomarkers, but no evident cognitive impairment reflecting very early disease. CSF levels of PSD-95 in these subjects were increased compared to HC and comparable to AD subjects with overt disease. PSD-95 levels were furthermore elevated in AD subjects with MCI compared to HC suggesting that increased CSF levels of PSD-95 are a phenomenon occurring early in AD as has been demonstrated for SNAP-25 and Ng. Further work can determine the degree to which PSD-95 levels reflect the stage or severity of the clinical syndrome of AD.

There is still an urgent need to identify drugs that can halt or reduce disease progression in AD, even with the recent advances in anti-amyloid treatment. The close association between cognitive decline and synaptic dysfunction renders synaptic proteins attractive for monitoring responses to disease-modifying drugs during clinical trials. The novel Simoa assays used in this study showed excellent performance in measuring CSF levels of PSD-95 and SNAP-25. The assays are performed on an easy to use and reliable automated platform that is widely available in AD biomarker labs and which allows for high throughput suitable for large-scale clinical trials. We used pre-commercial research versions of the assays where the two cohorts were measured using different lots which were manually prepared and did not go through the same level of lot-to-lot testing as standard commercial assays. This resulted in absolute differences in PSD-95 and SNAP-25 levels between the two cohorts, but with a tight correlation between a small number of bridge samples included in both cohorts indicating that the differences were due to a constant bias between the lots.

CSF biomarkers are attractive as CSF is in continuous direct exchange with the interstitial fluid of the brain. However, they are often difficult to implement especially in longitudinal studies where the invasiveness of the lumbar puncture limits repeat measurements. There is consequently a desire to identify biomarkers that can be detected in the blood. Robust blood biomarkers for synaptic pathology are, however, currently not available. Ng can be detected in the plasma, but levels do not correlate with CSF levels [28, 29] possibly due to peripheral expression of Ng [30]. SNAP-25 has not been detected in free form in the plasma but can be identified in neuron-derived exosomes isolated from serum with reduced levels in AD compared to HC [31]. Levels were furthermore correlated with the Mini Mental State Examination (MMSE) cognitive scores. Detection of CNS biomarkers in exosomes is, however, technically complex and expensive due to manual sample preparation and is not ready for larger studies. Our preliminary testing shows detectable levels of PSD-95 in plasma using the Simoa assay and future work will determine its diagnostic utility.

### Strengths and limitations

Strengths of this study include the robust assays used, parallel assessment of three synaptic proteins, replication of the main findings in a second cohort, and the range of diagnostic groups included. The subjects were well characterized by CSF AD biomarkers to avoid diagnostic misclassification and diagnoses were verified by a single experienced neurologist to provide consistency. Limitations include a lack of standardized neuropsychological testing and longitudinal follow-up, which precludes better evaluation of the relationship of the synaptic markers with cognition or prognosis. The large number of diagnostic groups included, many with varying age distribution, resulted in differences in average age between groups. Analysis of age effects did, however, not show any age effects on CSF levels of PSD-95 or SNAP-25. Differences between groups did also remain after controlling for age and sex.

## Conclusions

We conclude that AD subjects on average have higher CSF levels of PSD-95 and SNAP-25 compared to subjects with non-AD neurodegenerative diseases or other neurological conditions. High CSF levels of PSD-95 and SNAP-25 were, however, not specific for AD and were present in sporadic cases with inflammatory or vascular disorders as well. High CSF levels of PSD-95 were also observed in a few subjects with other neurodegenerative disorders. Together, these pre-and post-synaptic markers hold promise to identify early pathological abnormalities in AD, to correlate with cognitive decline, and to monitor responses to disease-modifying drugs reducing synaptic degeneration.

## Supplementary Information

**Additional file 1: Supplementary Figure 1.** Western blots of PSD-95 capture and detection antibodies. Homogenized brain samples were prepared by sonicating 100 mg of prefrontal cortex tissue from autopsy materials from two elderly subjects without cognitive complaints (Normal) and two subjects with Alzheimer’s disease (AD-Dem). Proteins were separated using sodium dodecyl sulphate-polyacrylamide gel electrophoresis (SDS-PAGE), transferred to a nitrocellulose membrane, and stained with the PSD-95 capture (anti-PSD-95 mouse IgG) or detection (anti-PSD-95 rabbit IgG) antibodies overnight at 4°C followed by fluorescently-conjugated secondary antibodies for 1h at RT in the dark. Imaging was performed using a Licor ODYSSEY CLx (LI-COR Biosciences, Lincoln, NE). A pre-stained precision plus protein dual color standards molecular weight ladder (10-250kDa; Bio-Rad, Hercules, CA) was used to estimate the molecular weight of the proteins analyzed. The western blots were performed in triplicate and repeated in two separate experiments to confirm that the results were reproducible.

**Additional file 2. Supplementary Figure 2.** Amyloid ß42/40 (Aß42/40) ratio in subjects with Alzheimer’s Disease (AD) and controls in (A) Cohort I measured using Quanterix Simoa Neurology 4-plex E (N4PE) assay and (B) Cohort II measured using Euroimmun Beta-Amyloid (1-40) and (1-42) ELISA assays. The cutoff for AD pathology was set at an Aß42/40 ratio of 0.065 in Cohort I and 0.080 in Cohort II (red dashed line). *Abbreviations*: HC=Healthy Controls, AD=Alzheimer’s Disease, MCI=Mild Cognitive Impairment, DEM=dementia, FTD=Frontotemporal Dementia, LBD/Park=Lewy Body Dementia/Parkinson’s Disease, MND=Motor Neuron Diseases, NOS=Not Otherwise Specified, INFL=Immune/Demyelinating Diseases, IIH=Idiopathic Intracranial Hypertension, NCtrl Other=Other Neurological Conditions, AD_Asympt=Cognitively unimpaired subjects with positive CSF AD biomarkers, NeuroDegen=Neurodegenerative Diseases.

**Additional file 3: Supplementary Table 1.** Correlations between CSF levels of synaptic markers in different groups.

**Additional file 4: Supplementary Table 2.** CSF levels of synaptic markers in diagnostic groups.

## Data Availability

The datasets used and/or analyzed during the current study are available from the corresponding author on reasonable request.

## Abbreviations

AUC: Area under the curve
CV: Coefficient of variation
Aß: Amyloid-ß
AD: Alzheimer’s disease
CNS: Central nervous system
CSF: Cerebrospinal fluid
GFAP: Glial fibrillary acidic protein
HC: Healthy control
LLOD: Lower limit of detection
LLOQ: Lower level of quantification
MCI: Mild cognitive impairment
NeuroDegen: Neurodegenerative diseases
NeuroCtrl: Neurological controls
NfL: Neurofilament light
Ng: Neurogranin
PSD-95: Postsynaptic density protein 95
pTau181: Phosphorylated-Tau(181)
SD: Standard deviation
SNAP-25: Synaptosomal-associated protein 25
