# Supplementary material for: Increased levels of the synaptic proteins PSD-95, SNAP-25, and neurogranin in the cerebrospinal fluid of patients with Alzheimer’s disease
Source: Alzheimers Res Ther. 2022 Apr 23;14:58. doi: 10.1186/s13195-022-01002-x (PMC9034610; doi:10.1186/s13195-022-01002-x)
Supplement: Supplementary file 1 — Additional file 1: Supplementary Figure 1. Western blots of PSD-95 capture and detection antibodies. Homogenized brain samples were prepared by sonicating 100 mg of prefrontal cortex tissue from autopsy materials from two elderly subjects without cognitive complaints (Normal) and two subjects with Alzheimer’s disease (AD-Dem). Proteins were separated using sodium dodecyl sulphate-polyacrylamide gel electrophoresis (SDS-PAGE), transferred to a nitrocellulose membrane, and stained with the PSD-95 capture (anti-PSD-95 mouse IgG) or detection (anti-PSD-95 rabbit IgG) antibodies overnight at 4°C followed by fluorescently-conjugated secondary antibodies for 1h at RT in the dark. Imaging was performed using a Licor ODYSSEY CLx (LI-COR Biosciences, Lincoln, NE). A pre-stained precision plus protein dual color standards molecular weight ladder (10-250kDa; Bio-Rad, Hercules, CA) was used to estimate the molecular weight of the proteins analyzed. The western blots were performed in triplicate and repeated in two separate experiments to confirm that the results were reproducible. [file 13195_2022_1002_MOESM1_ESM.pdf]

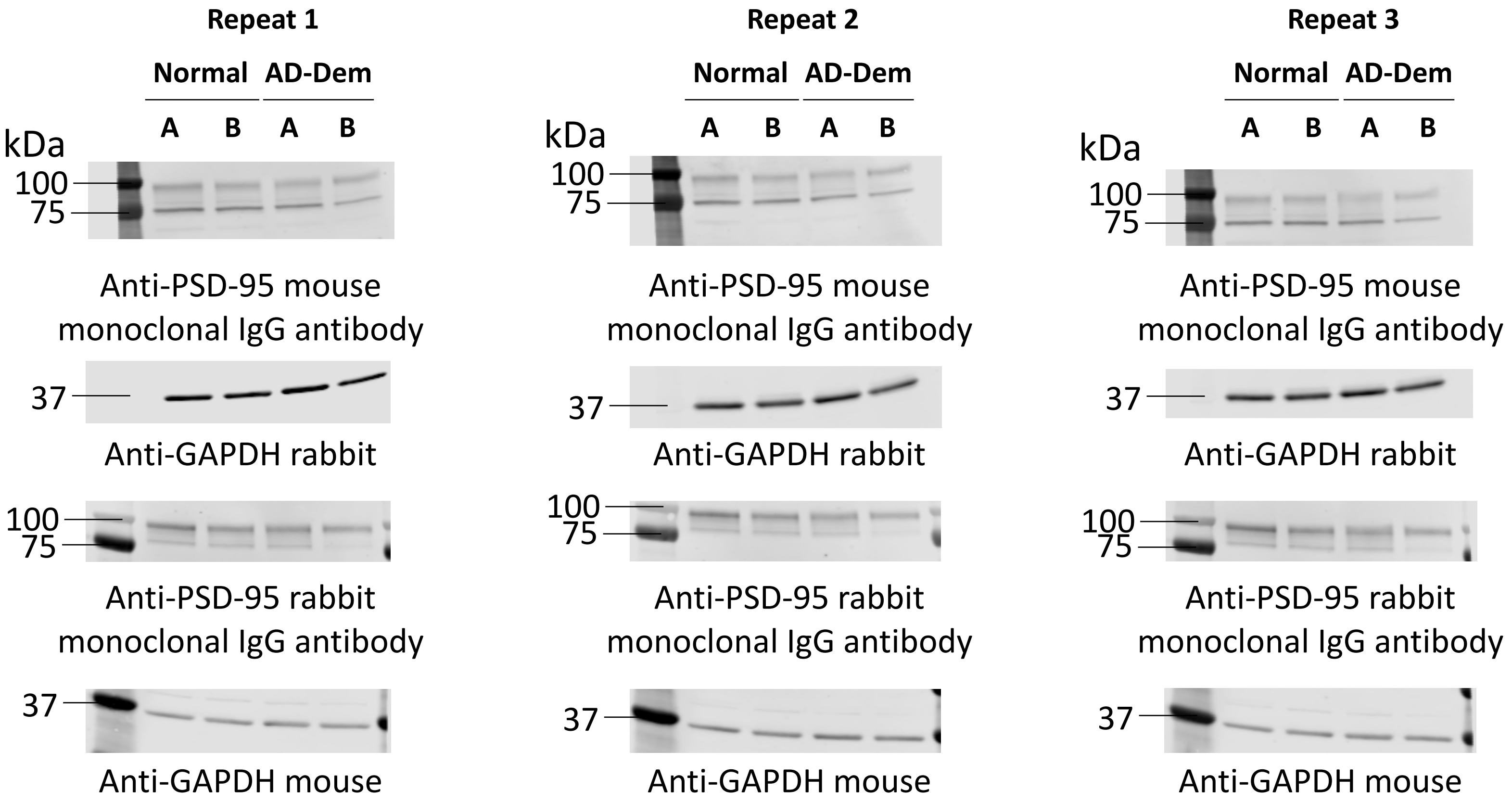

Supplementary Figure 1. Western blots of PSD-95 capture and detection antibodies. Homogenized brain samples were prepared by sonicating 100 mg of prefrontal cortex tissue from autopsy materials from two elderly subjects without cognitive complaints (Normal) and two subjects with Alzheimer's disease (AD-Dem). Proteins were separated using sodium dodecyl sulphate-polyacrylamide gel electrophoresis (SDS-PAGE), transferred to a nitrocellulose membrane, and stained with the PSD-95 capture (anti-PSD-95 mouse IgG) or detection (anti-PSD-95 rabbit IgG) antibodies overnight at 4°C followed by fluorescently-conjugated secondary antibodies for 1h at RT in the dark. Imaging was performed using a Licor ODYSSEY CLx (LI-COR Biosciences, Lincoln, NE). A pre-stained precision plus protein dual color standards molecular weight ladder (10-250kDa; Bio-Rad, Hercules, CA) was used to estimate the molecular weight of the proteins analyzed. The western blots were performed in triplicate and repeated in two separate experiments to confirm that the results were reproducible.
