# Supplementary figures and images for: Increased levels of the synaptic proteins PSD-95, SNAP-25, and neurogranin in the cerebrospinal fluid of patients with Alzheimer’s disease
Source: Alzheimers Res Ther. 2022 Apr 23;14:58. doi: 10.1186/s13195-022-01002-x (PMC9034610; doi:10.1186/s13195-022-01002-x)

A

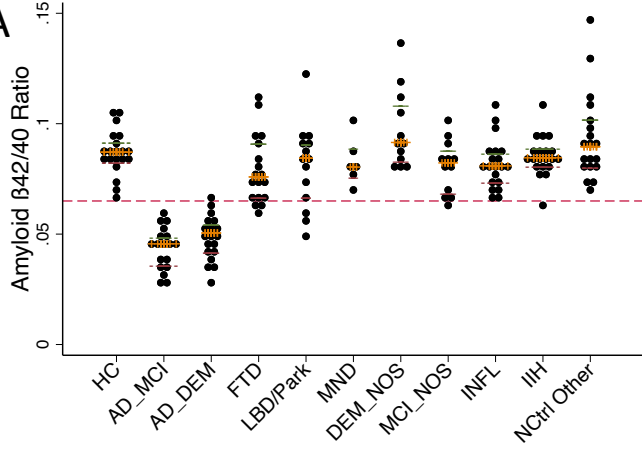

B

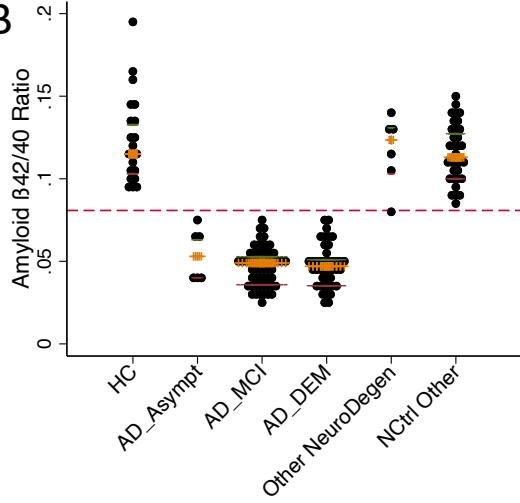

Supplement: Supplementary file 2 — Additional file 2. Supplementary Figure 2. Amyloid ß42/40 (Aß42/40) ratio in subjects with Alzheimer’s Disease (AD) and controls in (A) Cohort I measured using Quanterix Simoa Neurology 4-plex E (N4PE) assay and (B) Cohort II measured using Euroimmun Beta-Amyloid (1-40) and (1-42) ELISA assays. The cutoff for AD pathology was set at an Aß42/40 ratio of 0.065 in Cohort I and 0.080 in Cohort II (red dashed line). Abbreviations: HC=Healthy Controls, AD=Alzheimer’s Disease, MCI=Mild Cognitive Impairment, DEM=dementia, FTD=Frontotemporal Dementia, LBD/Park=Lewy Body Dementia/Parkinson’s Disease, MND=Motor Neuron Diseases, NOS=Not Otherwise Specified, INFL=Immune/Demyelinating Diseases, IIH=Idiopathic Intracranial Hypertension, NCtrl Other=Other Neurological Conditions, AD_Asympt=Cognitively unimpaired subjects with positive CSF AD biomarkers, NeuroDegen=Neurodegenerative Diseases. [file 13195_2022_1002_MOESM2_ESM.pdf]
